# Shift from stochastic to spatially-ordered expression of serine-glycine synthesis enzymes in 3D microtumors

**DOI:** 10.1038/s41598-018-27266-8

**Published:** 2018-06-20

**Authors:** Manjulata Singh, Katsuhiko Warita, Tomoko Warita, James R. Faeder, Robin E. C. Lee, Shilpa Sant, Zoltán N. Oltvai

**Affiliations:** 1 Department of Pharmaceutical Sciences, School of Pharmacy, University of Pittsburgh, Pittsburgh, PA 15261 USA; 2 Department of Pathology, School of Medicine, University of Pittsburgh, Pittsburgh, PA 15213 USA; 3 Department of Computational & Systems Biology, School of Medicine, University of Pittsburgh, Pittsburgh, PA 15260 USA; 4 Department of Bioengineering, Swanson School of Engineering, McGowan Institute for Regenerative Medicine, and UPMC-Hillman Cancer Center, University of Pittsburgh, Pittsburgh, PA 15261 USA

## Abstract

Cell-to-cell differences in protein expression in normal tissues and tumors are a common phenomenon, but the underlying principles that govern this heterogeneity are largely unknown. Here, we show that in monolayer cancer cell-line cultures, the expression of the five metabolic enzymes of serine-glycine synthesis (SGS), including its rate-limiting enzyme, phosphoglycerate dehydrogenase (PHGDH), displays stochastic cell-to-cell variation. By contrast, in cancer cell line-derived three-dimensional (3D) microtumors PHGDH expression is restricted to the outermost part of the microtumors’ outer proliferative cell layer, while the four other SGS enzymes display near uniform expression throughout the microtumor. A mathematical model suggests that metabolic stress in the microtumor core activates factors that restrict PHGDH expression. Thus, intracellular enzyme expression in growing cell ecosystems can shift to spatially ordered patterns in 3D structured environments due to emergent cell-cell communication, with potential implications for the design of effective anti-metabolic cancer therapies.

## Introduction

In nature, most cells exist as part of a cellular ecosystem, whether it is bacterial biofilms, tissue and tumor ecosystems, or highly organized tissue architectures. Cells of the same type, but in different positions in their ecosystem, may have different metabolism and function due to signals from neighboring cells and from the local microenvironment. Indeed, tissue culture studies have demonstrated widespread protein expression heterogeneity in two-dimensional (2D) monoclonal cell cultures^1–3^, indicating phenotypic variability in cell function^4^. Similarly, the human protein atlas reveals that most metabolic enzymes display spatially variable expression in most tumor types^5,6^. The observed enzyme expression heterogeneity may reflect the tumor cells’ response to signals from their local environment both, due to nutrient- and/or oxygen gradients or due to autocrine- or non-cell autonomous paracrine effects from other tumor cells or non-tumor cell types^7^. The influence of these factors on the system-level organization of cell function, including cell metabolism remains only partially understood.

**Figure 1:**
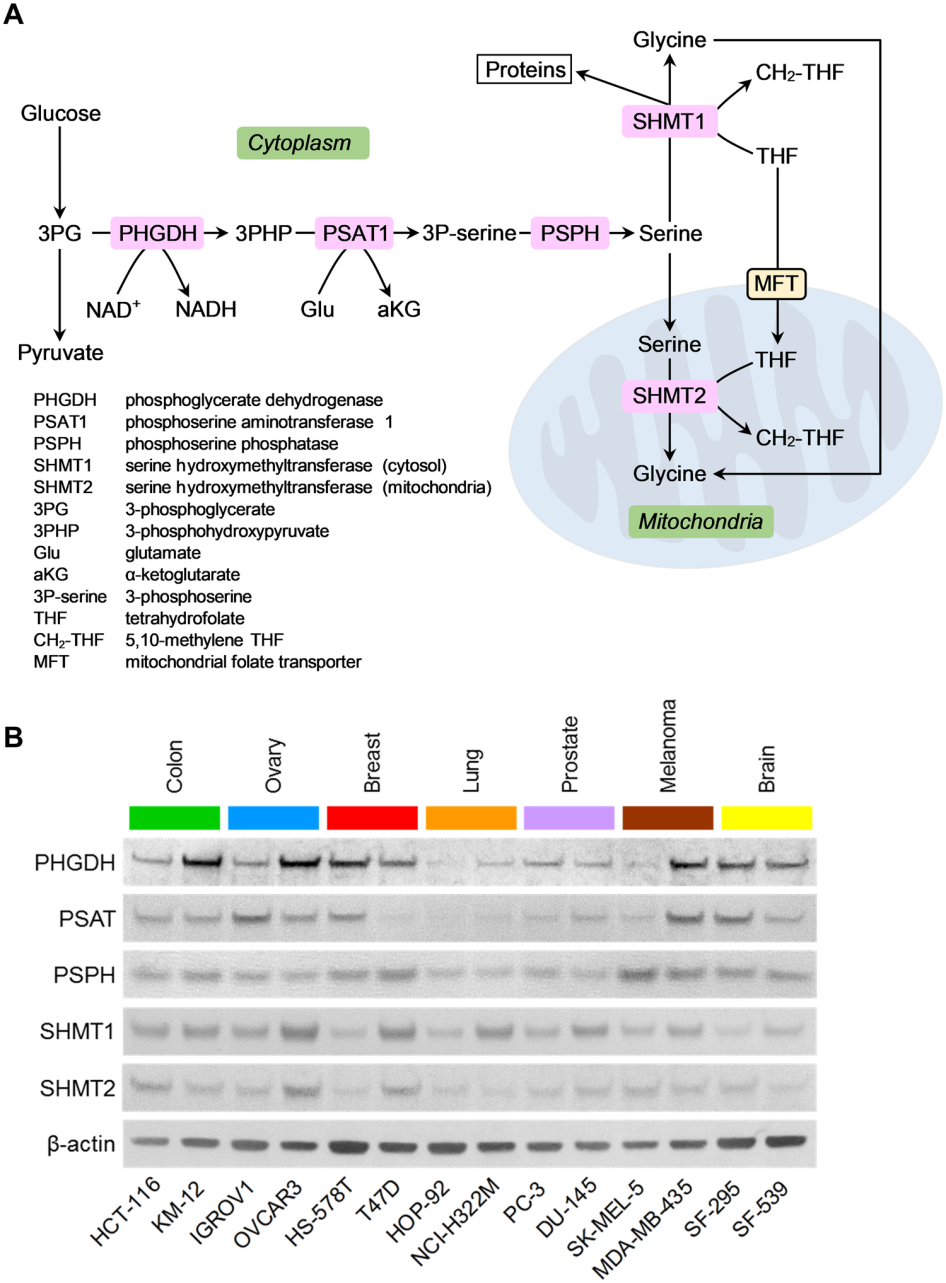
Average expression levels of serine-glycine synthesis enzymes in cancer cell lines. (**A**) Schematic representation of pathways of serine-glycine synthesis and one-carbon metabolism. The enzymes examined in this study are highlighted with pink boxes, while cellular compartments are highlighted with green boxes. (**B**) Immunoblots of serine-glycine synthesis pathway enzymes in the indicated fourteen human cancer cell lines derived from seven human tissues are shown. Βeta-actin was used as loading control. The grouping of immunoblots were obtained from cropped blots obtained from different gels and are delineated by white space between them.

Metabolic models suggest that above a threshold ATP and/or biomass production (cell division) rate, cells switch from oxidative phosphorylation (OxPhos) to overflow metabolism (i.e., mixed OxPhos/fermentation)^8–10^, which could explain the observed differences between rapidly proliferating and slowly dividing sectors of a growing tumor. This metabolic reorganization is predicted to involve upregulation of the serine-glycine synthesis and one-carbon metabolism (SGOC) pathways^11^ (Fig. 1A). Experimental data indicate the enhanced activity of these pathways in rapidly proliferating tumors, embryonic stem cells and cancer cell lines^12–16^, which support both anabolic and catabolic processes.

Three-dimensional (3D) culture systems are physiologically more relevant and often show differential gene expression and drug responses as compared to 2D cell monolayers^17,18^. Moreover, various pre-clinical studies suggest that 2D monolayer cultures sometimes fail to predict *in vivo* drug responses^19^. We have recently developed a hydrogel microwell platform to generate hundreds of uniform, discrete-sized, 3D *in vitro* microtumors using a variety of cancer cell lines (breast, head and neck cancer, and lung) and primary patient-derived cells (breast cancer, mesothelioma)^18,20^. Precise control of microtumor size is expected to create spatial oxygen/nutrient diffusion gradients leading to controlled yet reproducible local microenvironments. Indeed, without any external stimulus, microtumors derived from select cancer cell lines develop three key hallmarks of tumor progression observed *in vivo*: increasing microtumor size drives hypoxia and metabolic stress; heterogeneous tumor cells expressing different levels of E-cadherin (epithelial marker) and vimentin (mesenchymal marker) spontaneously emerge; and peripheral cells begin to migrate from the parent tumor. A tangible advantage of our microtumor platform is the ability to study precisely and reproducibly how the emergent microenvironment induces tumor cell heterogeneity in isolation from non-tumor cells present *in vivo*, providing a unique opportunity to define tumor-intrinsic mechanisms of emergence of intratumoral heterogeneity in expression levels of different proteins.

Here, we examine the expression of the five metabolic enzymes of serine-glycine synthesis (SGS) (Fig. 1A) in two different cell ecosystems derived from the same monoclonal tumor cell line. We show that in 2D cell cultures, PHGDH expression is stochastic and does not strictly correlate with cell proliferation, as indicated by the Ki-67 expression status of cells. By contrast, in DU-145 cancer cell line-derived 3D microtumors, PHGDH expression is restricted to the outermost part of the Ki-67^+^ proliferative outer layer of the microtumors in direct contact with the culture medium, while expression levels for the other four SGS enzymes are distributed more uniformly throughout. A mathematical model suggests that emergent metabolic stress in the core of the growing microtumor regulates factor(s) that restrict the expression of PHGDH. Thus, intracellular enzyme expression in monoclonal cell ecosystems can shift from stochastic to a spatially organized pattern when cells encounter 3D structured microenvironments, in which tight regulation of spatial expression may be limited to rate-limiting enzymes of the metabolic pathways.

## Results

### Metabolic enzymes of the serine-glycine synthesis pathway display variable average expression in 2D cell monolayer cultures

Variability of serine and glycine uptake between tumor cell lines^21,22^ indicate that cell lines may synthesize amino acids with different rates, and suggest that expression levels of their SGOC pathway enzymes, including those of serine-glycine synthesis (SGS) may also differ. To test this hypothesis, we selected a panel of fourteen human cancer cell lines from the NCI-60 cancer cell line panel^23^ representing seven different human tumor types that possess various average cell volumes, cell protein amount and protein concentrations per cell^22^ or position on the epithelial-to-mesenchymal transition spectrum^24^. We then determined the average cellular expression levels of the SGS enzymes, phosphoglycerate dehydrogenase (PHGDH), phosphoserine aminotransferase (PSAT), phosphoserine phosphatase (PSPH) and serine hydroxymethyltransferases 1 and 2 (SHMT1, SHMT2) (Fig. 1A) when these cells were propagated in serine and glycine-containing (complete) growth medium. Using immunoblotting, we found that the average enzyme expression levels vary significantly among these cell lines; Several cancer cell lines expressed PHGDH at high levels, while other cell lines displayed low to minimal PHGDH expression (Fig. 1B). We observed similar variations in average PSAT, PSPH, SHMT1 and SHMT2 expression levels (Fig. 1B) that did not show strong correlations with the doubling times of these cell lines (Fig. S1). Thus, when grown as 2D monolayers, tumor cell lines display variable average expression levels of SGS enzymes that do not trivially correlate with their cell proliferation rate.

### Stochastic expression of serine-glycine synthesis enzymes in tumor cell monolayers

**Figure 2:**
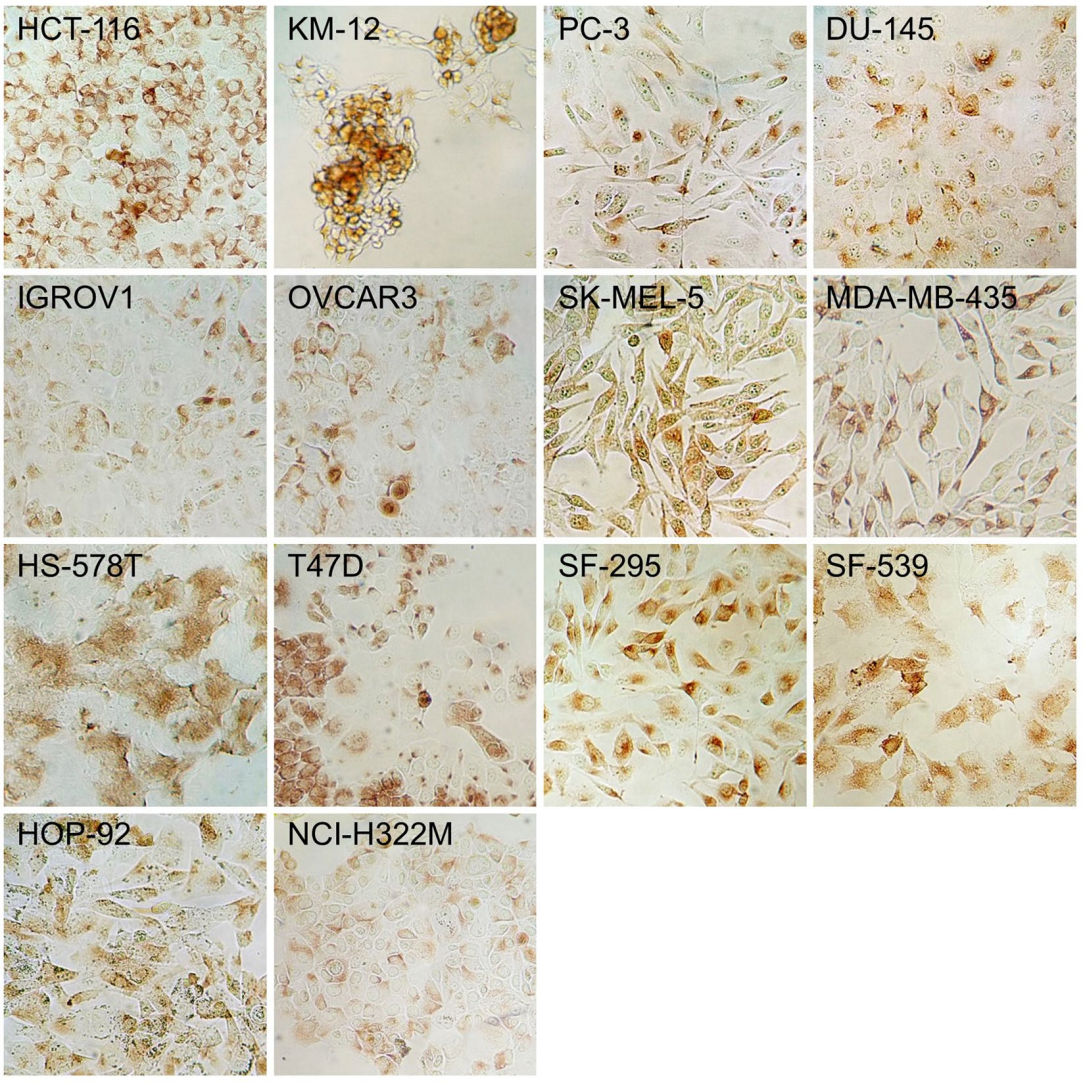
PHGDH expression in monolayer cultures of fourteen NCI-60 cell lines. PHGDH immunoreactivity of colon cancer (HCT-116 and KM-12), ovarian cancer (IGROV1 and OVCAR3), breast cancer (HS-578T and T47D), lung cancer (HOP-92 and NCI-H322M), prostate cancer (PC-3 and DU-145), melanoma (SK-MEL-5 and MDA-MB-435), and brain cancer (SF-295 and SF-539) derived cell lines from the NCI-60 collection is observed in tumor cell with various expression levels and immunoreactive areas. 3,3′-diaminobenzidine was used for visualization.

Expression levels of proteins are often highly heterogeneous even among cells of isogenic and phenotypically uniform cell lines^25,26^. To determine if cell-cell expression variability also exists for SGS enzymes, we first studied the expression characteristics of PHGDH, the first enzyme of the serine synthesis pathway (Fig. 1A). Under standard 2D monolayer growth conditions, and employing immunocytochemistry-based testing, we have found that cytoplasmic PHGDH expression varies randomly among cells of all fourteen cell lines, some cells expressing PHGDH at high level while others do so at substantially lower levels (or not at all) (Fig. 2). As PHGDH is the rate-limiting enzyme of serine synthesis, this finding implies substantial cell-to-cell variability in the level of *de novo* serine biosynthesis from the glycolytic intermediate, 3-phosphoglycerate (3PG) (Fig. 1A) in each cancer cell line when grown in complete growth medium.

To examine further the characteristics of PHGDH expression, we created limiting dilutions of two of the cell lines, the DU-145 and PC-3 prostate cancer-derived cell lines. Single cell-derived clones of these cell lines displayed nearly homogeneous PHGDH expression (ON) or minimal expression (OFF) (Fig. S2), suggesting that stochastic PHGDH ON/OFF expression is maintained in the individual clones for several generations. Such stochastic protein expression pattern may be common for many proteins in cells of 2D monolayers.

Heterogeneous PHGDH expression suggests that the two other serine synthesis enzymes may also display similar cell-to-cell variability in their expression level. Therefore, we next performed dual immunofluorescence staining to determine the paired expression of PHGDH and PSAT, or PHGDH and PSPH in tumor cell monolayers. Single cells in monolayers of DU-145 and PC-3 cell lines displayed variable levels of expression for PHGDH, PSAT and PSPH, having either high, intermediate or low/very low amounts of each enzyme. We also find substantial variability in the relative expression ratios of PHGDH to PSAT and PHGDH to PSPH within individual cells with some cells having relatively high PSAT/PHGDH and PSPH/PHGDH ratios, while other cells displayed the opposite pattern (Fig. S3). These findings indicate that in 2D monolayer cultures, these enzymes display substantial cell-cell variability in their expression and relative expression ratios in individual cells.

In contrast, DU-145 and PC-3 cell monolayers display less cell-to-cell heterogeneity in the expression of cytoplasmic SHMT1 and mitochondrial SHMT2 (Figs. S4 and S5). These findings indicate that in 2D monolayer cultures, these cell lines display less variability in the expression of serine-glycine interconversion enzymes than for the enzymes of glucose-derived serine synthesis.

### PHGDH expression does not strongly correlate with Ki-67 expression in monolayer cultures

**Figure 3:**
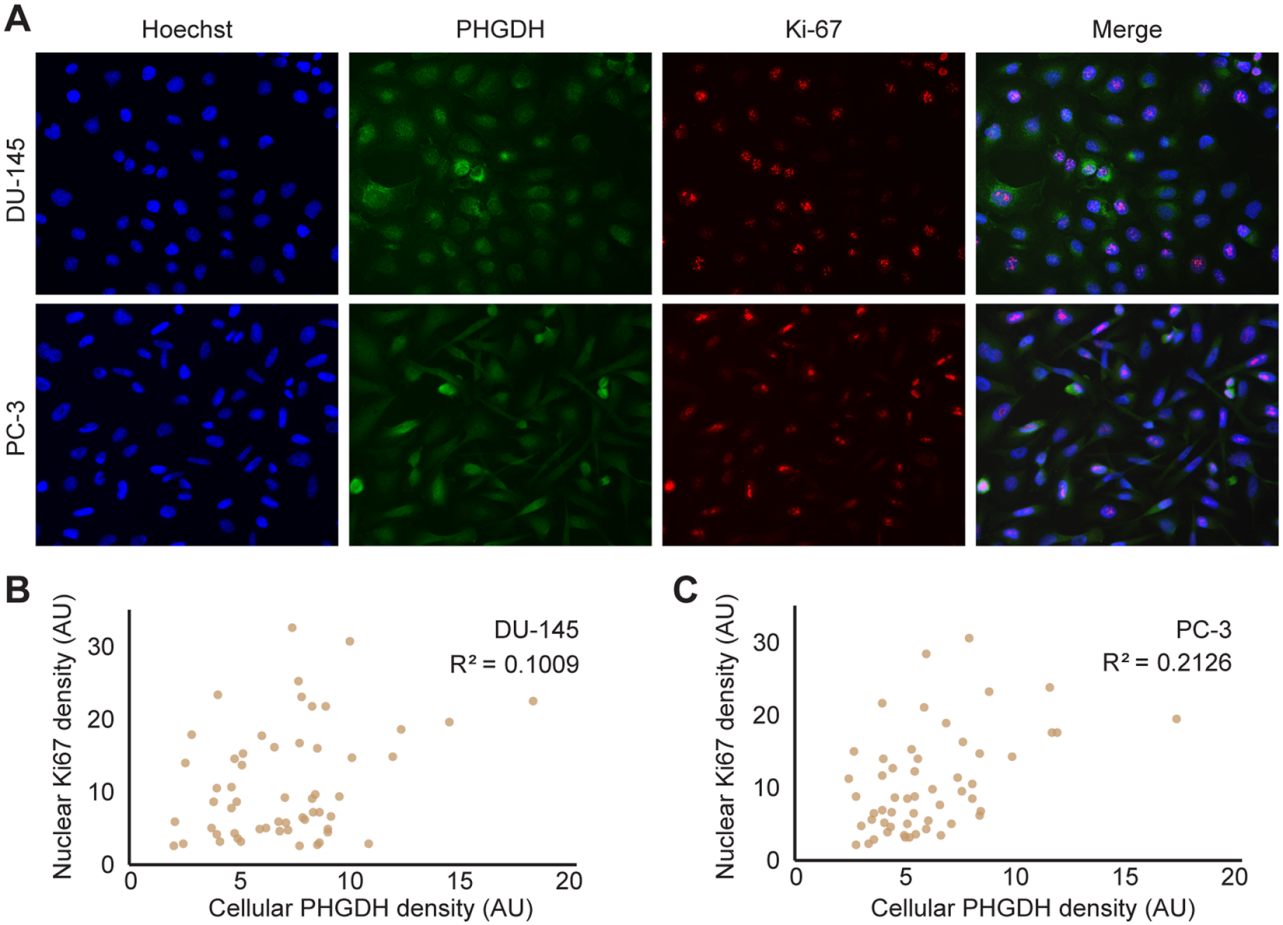
Expression of PHGDH and Ki-67 in DU-145 and PC-3 cell line monolayers. (**A**) Cytoplasmic PHGDH (green channel), and nuclear Ki-67 (red channel) immunoreactivity are shown in DU-145 and PC-3 prostate cancer cell lines. Hoechst counterstain (blue color) indicates cell nuclei. Scatter plots for fluorescence of nuclear Ki67 versus cellular PHGDH quantified from images of single cells shown in for (**B**) DU-145 and (**C**) PC-3 cells.

Previous metabolic modeling results have suggested that above a threshold growth rate, cells switch from oxidative phosphorylation (OxPhos) to overflow metabolism (i.e., to mixed Oxphos/ aerobic fermentation)^8–10^. The predicted metabolic reorganization involves upregulation of the serine-glycine synthesis and one-carbon metabolism (SGOC) pathways^11^ (Fig. 1A). Recent experimental data indicate the activity of this pathway in rapidly proliferating tumors, embryonic stem cells and cancer cell lines that support both anabolic and catabolic processes^13,15,27^. We therefore, next examined if PHGDH expression correlates with cell proliferation. To this end, we co-immunostained cells for their PHGDH expression and for the expression of the cell proliferation marker, Ki-67, which shows nuclear expression throughout the cell cycle (Fig. 3A). Dual staining for PHGDH and Ki-67 revealed cell-to-cell variability in their expression levels and only partial overlap (Fig. 3A, right panels) and limited correlation in the cellular expression of the two proteins (Fig. 3B,C), some cells expressing only one of the proteins while some expressing both (Fig. 3A, right panels). Assuming that PHGDH expression indicates cell-autonomous serine synthesis, then cell-autonomous serine synthesis is not uniformly required for cancer cell proliferation in 2D monolayer cultures in complete growth medium.

### Spatially organized heterogeneity of PHGDH expression in 3D microtumors

Cell-to-cell heterogeneity in the expression of serine synthesis enzymes in 2D monolayer cultures may indicate a population-level “bet hedging” mechanism^28–30^, a strategy that ensures at least a sub-population of cells will respond appropriately to environmental challenges. In the context of a 3D tumor, we hypothesized that the spatial organization of cells can influence local environmental signals or heterotypic interactions between cells within different regions of the tumor, and thereby constrain the amount of expression variability. For example, the central region of a tumor mass is often deprived of oxygen and, consequently, expression of SHMT2 is essential to maintain proper redox balance in hypoxic regions of the tumor^31^.

**Figure 4:**
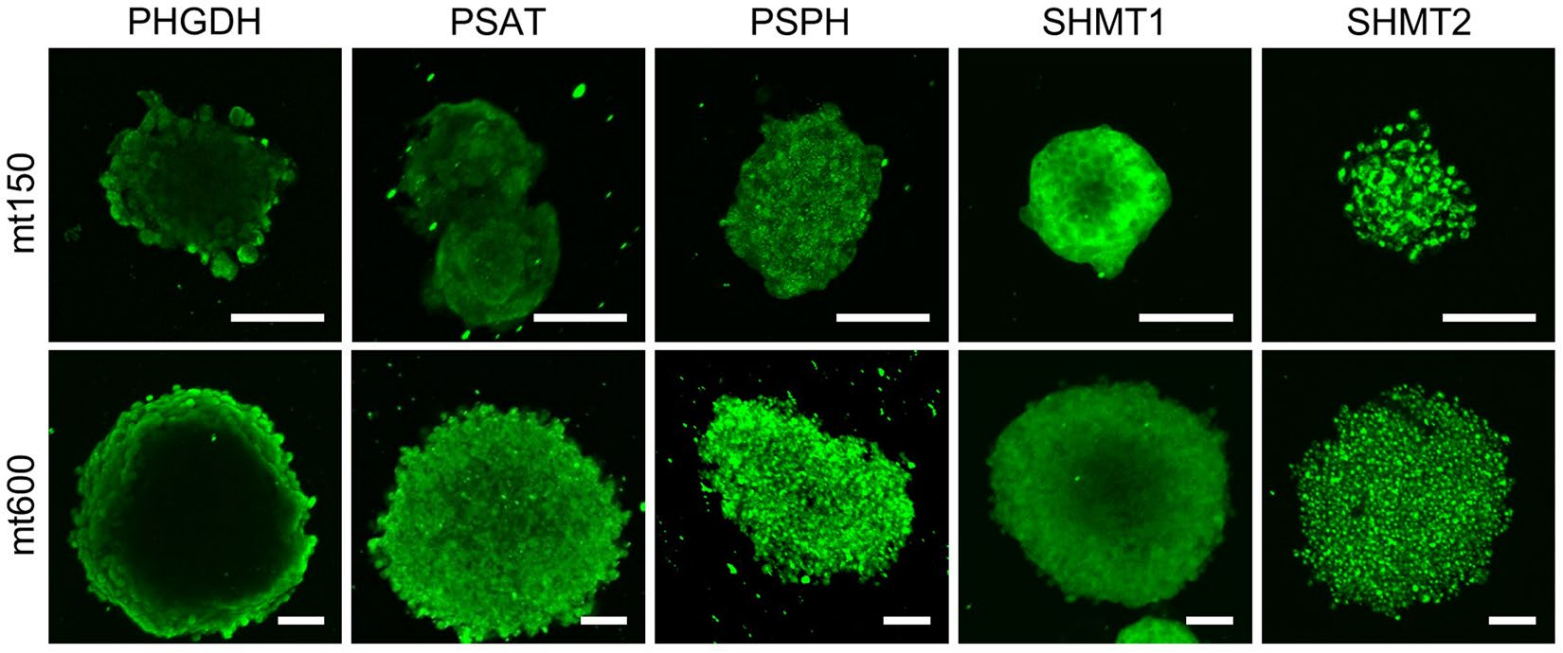
Spatial localization of serine-glycine synthesis enzymes expression in 3D microtumors. Spatial localization of the indicated enzymes in mt150 (top panels) and mt600 (bottom panels) DU-145-derived 3D-microtumors. Confocal images are centered on the middle plane of the microtumor. Bar = 100 μm.

To test this hypothesis and to examine whether expression patterns for metabolic enzymes are affected by microtumor size, we established 3D microtumors from DU-145 cells using a previously described hydrogel array^18,20^ (Fig. S6). We chose to use two different size of microtumors; small, relatively non-hypoxic, 150 µm in diameter and hypoxic 600 µm in diameter (Fig. S7), which are henceforth referred to as mt150 and mt600, respectively. (We also attempted to culture PC-3 cells in the same way; However, due to their highly mesenchymal nature^24^ and rapid migration out of the wells (not shown) we could not establish compact 3D microtumors for this cell line.) We then analyzed the expression levels for SGS enzymes in mt150 and mt600 DU-145 cell line-derived microtumors. We have found that in mt150 and mt600 microtumors PHGDH expression is highly spatially organized and is restricted to the periphery of the microtumor mass irrespective of their size (Figs. 4, S8). By contrast, the second enzyme of the pathway, PSAT displayed a more uniform expression that was at times stronger on the periphery of the tumor mass in both mt150 and mt600 microtumors compared to central core (Figs. 4, S9A). Finally, the third enzyme of the pathway, PSPH, displayed uniform expression in both mt150 and mt600 microtumors (Figs. 4, S9B). As PHGDH is the rate limiting enzyme of 3-phosphoglycerate-derived serine synthesis, these data indicate that in isogenic DU-145 cell-derived microtumors, in complete growth medium 3PG-derived serine synthesis (Fig. 1A) is restricted to the periphery of the microtumors’ mass.

Reversible conversion of serine to glycine is achieved through the activity of cytoplasmic SHMT1 and mitochondrial SHMT2 activities (Fig. 1A). We found that in DU-145 cell line-derived mt150 and mt600 microtumors, SHMT1 displayed a wide, doughnut-shaped cytoplasmic enzyme expression pattern with weaker expression in their cores (Fig. 4). By contrast, SHMT2 expression was uniform throughout in both microtumor types (Fig. 4) and co-localized with a mitochondrial marker protein, TOM-20 (Fig. S10), confirming its mitochondrial localization.

### PHGDH expression partially overlaps with the zone of cell proliferation in 3D microtumors

**Figure 5:**
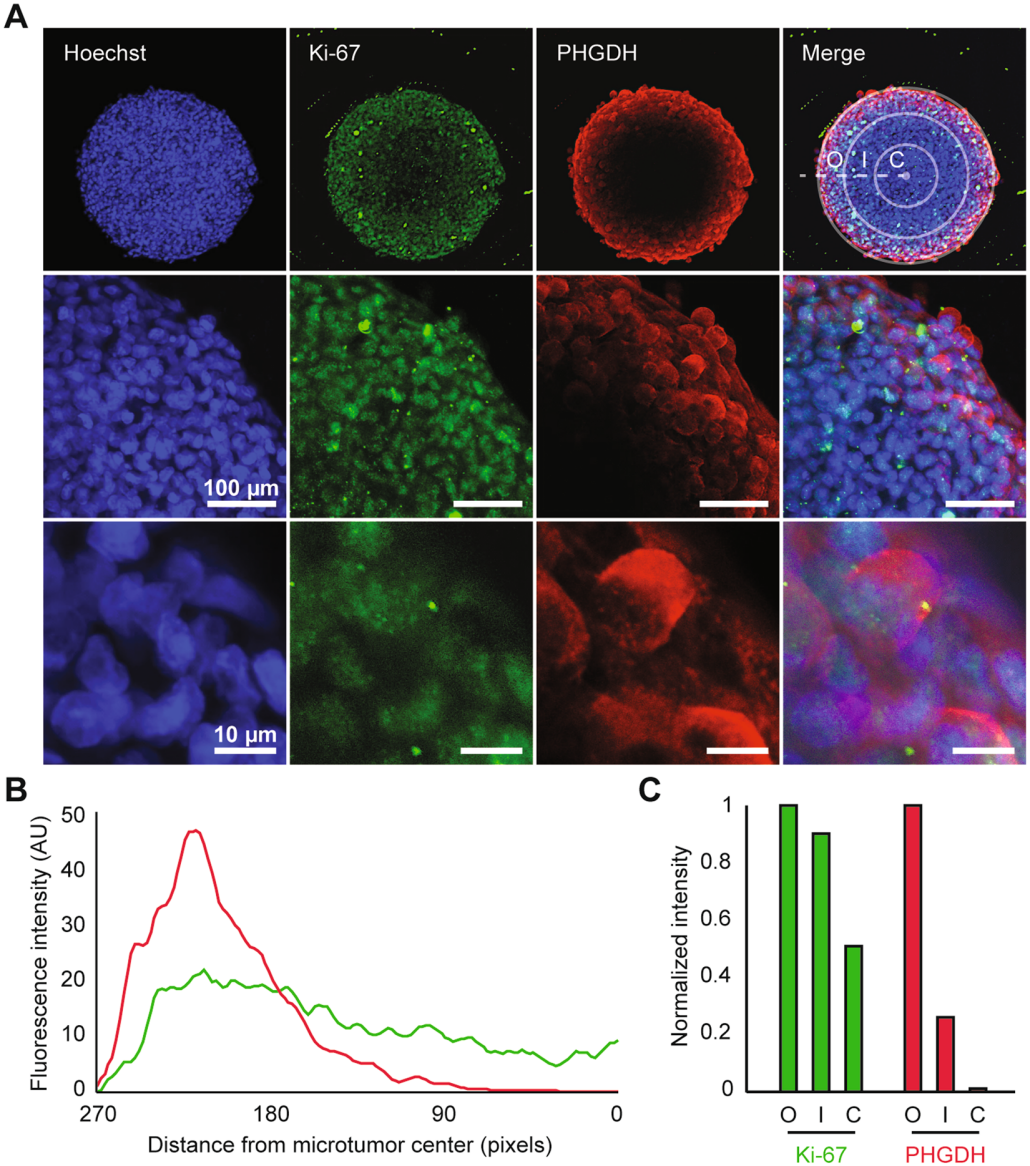
PHGDH and Ki-67 expression in 3D microtumors. (**A**) Spatial localization of Ki-67 (green), PHGDH (red), and their overlap in mt600 microtumors at low- (top panel), intermediate (middle panel) and high magnification (bottom panel). Confocal images are centered on the middle plane of the microtumor. In the overlay panels Hoechst counterstain (blue color) indicates cell nuclei. PHGDH expression is restricted to the outer layer of Ki-67^+^ cells. Concentric circles in the top right panel depict the outer layer (O), inner layer (I), and core (C) regions of the microtumor. (**B**) Average of nine radial linescans for Ki-67 (green) and PHGDH (red) fluorescence intensity. (**C**) Average of Ki-67 and PHGDH fluorescence intensity in each of the microtumor compartments.

Empirical data indicate that the serine synthesis pathway is upregulated in rapidly proliferating cells^12–16^. Therefore, we analyzed the expression of PHGDH in relation to the proliferation marker, Ki-67. In mt600 microtumors the expression of both Ki-67 and PHGDH proved highly spatially organized, proliferating Ki-67^+^ cells forming a broad outer layer of the growing microtumor (Fig. 5A). In contrast, PHGDH expression proved more restricted to the outermost cell layers (Fig. 5A), in which normoxia is evident (Fig. S7). Radial linescans (Fig. 5B) have identified varied levels of average Ki-67 and PHGDH fluorescence intensity in each of the microtumor compartments (Fig. 5C). These data demonstrate the existence of two separate layers of proliferating cells, a PHGDH positive outermost layer (O) and a PHGDH low positive inner layer (I) of proliferating cells. The two proliferative layers in turn encompass a non-proliferating (PHGDH negative/ Ki-67 low expression) microtumor core (C) (Fig. 5A–C). Thus, in serine-containing growth medium, glucose-derived *de novo* serine synthesis appears to be required for cell growth and proliferation in the outermost cell layers of the microtumors.

### Emergent gradients of nutrients and stress signals emanating from the microtumor’s core may regulate spatial protein expression in 3D microtumors

**Figure 6:**
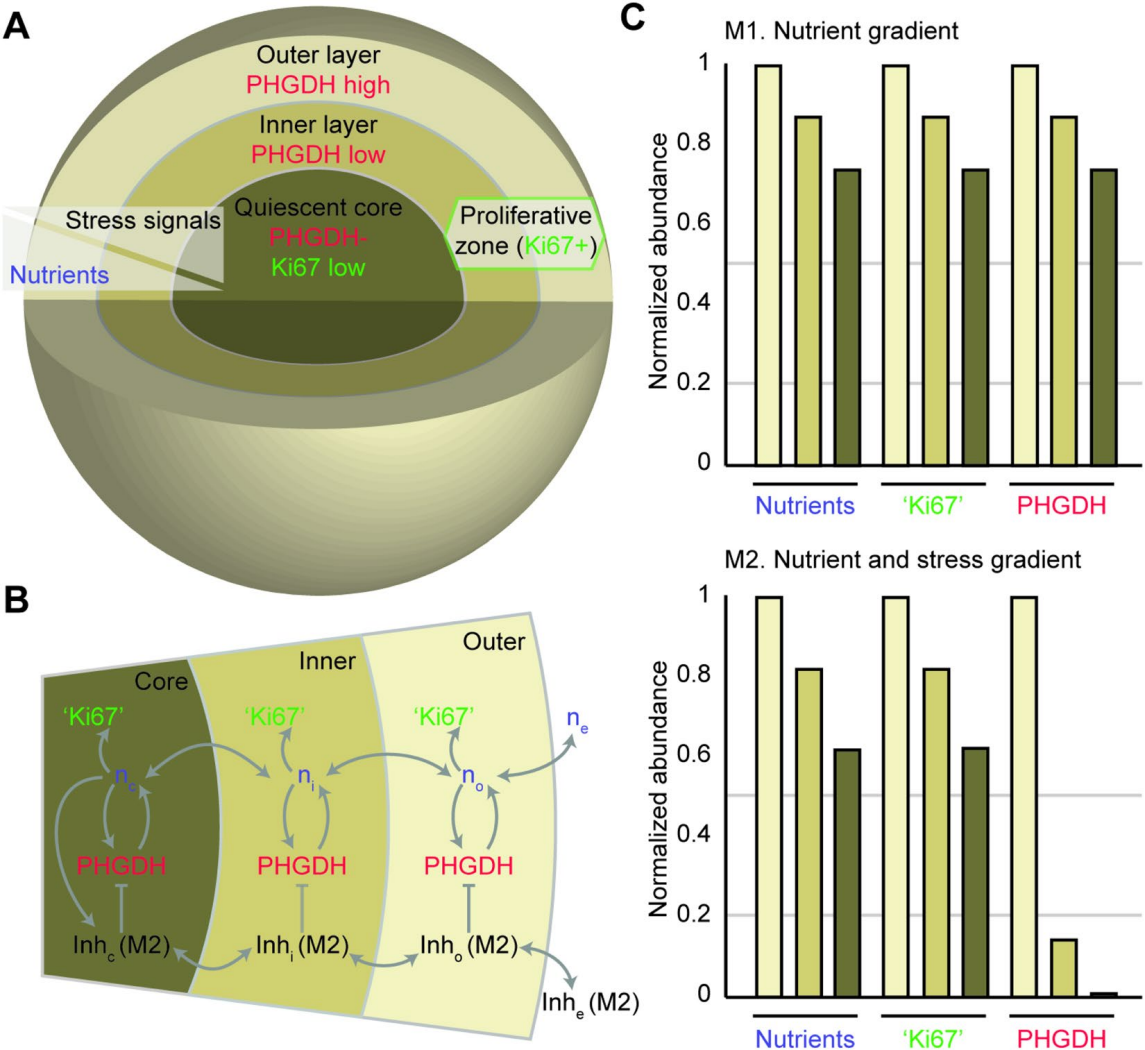
Spatial organization in a compartmental model of 3D microtumors. (**A**) Compartments of a 3D microtumor model include an outer layer that separates an inner layer and core of tumor cells from the extracellular compartment. A decreasing gradient of nutrients in models 1 and 2 (M1 and M2, respectively) is formed through nutrient consumption and diffusion between the outer layer, inner layer, and core of the microtumor. M2 also considers a gradient of stress signals that are strongest at the microtumor core. (**B**) Schematic diagram of the transport of nutrients (n) and their consumption for the expression of proliferative genes associated with Ki-67^+^ status (‘Ki67’), the rate-limiting enzyme for serine biosynthesis (PHGDH), and a molecule that inhibits expression of PHGDH (Inh). Expression of Inh is omitted in M1. Extracellular, outer layer, inner layer, and core compartments are noted in subscripts (e, i, o, and c respectively). (**C**) The abundance of nutrients, ‘Ki67′, and PHGDH in each of the microtumor compartments for M1 (top) and M2 (bottom). Each quantity is normalized to its abundance in the outer layer compartment.

To gain insight into mechanism(s) that may spatially restrict PHGDH and Ki-67 expression in 3D microtumors, we developed a multi-compartment mathematical model. The model assumes that nutrients diffuse from the growth medium (i.e., extracellular compartment) towards the core of the microtumor through two intervening layers (Fig. 6A). Available nutrients in each layer of the microtumor are either consumed for production of biomass, including PHGDH and proliferative factors that contribute to Ki-67^+^ status (referred to as ‘Ki67′ in simulations), or transported between layers (Fig. 6B). PHGDH expressed in each layer also contributes to the pool of available nutrients through *de novo* amino acid biosynthesis. In all simulations, the abundance of nutrients formed a decreasing gradient from the nutrient-rich outer layer towards the nutrient-starved core (Fig. 6C). Because the production of proliferative factors and PHGDH are both dependent on nutrient availability only, the abundance of ‘Ki67′ and PHGDH relative to the outer layer are exactly correlated throughout the microtumor (Fig. 6C, top). Although simulation results from our model (M1) that assumes only a decreasing nutrient gradient toward the microtumors’ core are consistent with the pattern of Ki-67 from immunofluorescence images of 3D microtumors, they contrast with the pattern of expression observed for PHGDH (Fig. 5A–C).

The core of 3D microtumors mimic several defining characteristics of tumors *in vivo*, including increased acidity and reduced concentrations of nutrients and oxygen that leads to remodeled expression of many metabolic and non-metabolic genes. We reasoned that metabolic stresses from the microtumor core may therefore provide additional regulatory signals that restrict PHGDH expression within microtumor compartments. To simulate the aggregate effects of stress-dependent factors on PHGDH expression, a second model (M2) included a hypothetical inhibitory molecule expressed in the microtumor core (Fig. 6C, bottom). An inhibition gradient with direction opposite to the nutrient gradient forms when the molecule can transport between compartments (Fig. 6B) or when the inner proliferative layer of the microtumor experiences the same metabolic stress as the core, but to a lesser extent. In results from M2, distributions for the abundance of nutrients and ‘Ki67′ were comparable to previous simulations; however, the relative abundance of PHGDH was strongly reduced in the inner layer and core compartments (Fig. 6C, bottom), as seen in the experiments. Together, our simulations suggest that localized stresses within a 3D microtumor may lead to emergent cell-cell communication and contribute to the spatial organization of metabolic enzyme expression.

## Discussion

Tumors are complex ecosystems in which spatiotemporal interactions among malignant, stromal and immune cells and self-deposited extracellular matrix collectively define their biological behavior. Genomic heterogeneity is widespread in tumors^32^, and recent work also indicate substantial transcriptomic heterogeneity in various tumor types^33,34^. Molecular heterogeneity is evident on proteomic^1–3^ and metabolic levels^35–37^, as well, attributed in part to the tumors’ tissue of origin^38,39^ and to emergent signaling interactions among cancer- and infiltrating normal cells in expanding tumors^7^. However, the cacophony of interactions among these cells, the dynamically molded microenvironment in growing tumors and interactions with self-deposited extracellular matrices makes it challenging to uncover the underlying principles that govern this multi-hierarchical expression heterogeneity.

One approach to overcome these limitations is to create synthetic tumors with precise control over their size and local microenvironments in which the various hallmarks of cancer, such as altered cell metabolism can be systematically examined^40–42^. Here, we have used cancer cell line-derived 3D microtumors as the simplest form of such synthetic tumor ecosystems and compared their expression profiles of five metabolic enzymes that catalyze serine-glycine synthesis in human cells to those seen in 2D monolayers. We have shown that, while the expression of PHGDH, the rate-limiting enzyme of serine synthesis from glucose, is stochastic in 2D monolayer cultures (Figs. 2 and 3), its expression is spatially restricted to the outermost cell layers in both small and larger microtumors (Figs. 4 and 5). This expression distribution partially overlaps with the Ki-67^+^ zone of active cell proliferation, but the latter extends radially toward the center of the microtumor (Fig. 5). By contrast, the expression of the two other serine synthesis enzymes in 3D microtumors is near uniform (Fig. 4). The expression of SHMT-2 is also uniform, while SHMT1 shows only slightly reduced expression in the core of microtumors (Fig. 4) indicating full activity of the mitochondrial and cytoplasmic arms of one-carbon cycle (Figs. 1A, S11) and maintenance of NADPH production and redox balance^31,43,44^. This finding also hints at an unexpected regulatory hierarchy, in which the expression of rate-limiting enzymes such as PHGDH are strictly controlled, while the other enzymes of the pathways are likely only controlled post-translationally.

What drives the observed shift in PHGDH expression pattern in 3D microtumors? Our modeling indicates that this may be an emergent property from the opposing gradients of nutrient and oxygen limitation and of a signaling molecule produced by cells of the stressed microtumor core (Fig. 6). By contrast, in 2D monolayer cultures cells do not encounter either of these gradients. The seemingly stochastic enzyme expressions seen in fixed cells of 2D cultures may mask unsynchronized oscillations (or more complex time-varying dynamics) in enzyme expression levels that become synchronized or stabilized and spatially restricted when cells are in an emergent 3D structure. A similar scenario was demonstrated during the periodic segmentation of the presomitic mesoderm (PSM) during embryonic development where synchronized expression in PSM cells become unsynchronized when they are dissociated from their 3D environment^45^.

The fact that metabolic pathway activities, such as *de novo* serine synthesis display a spatially restricted pattern in 3D microtumors is not unexpected. Similar results have been seen in growing bacterial colonies that also display a rapidly proliferating outer layer with spatially restricted acetate metabolism that is predicted to contribute to crosstalk among the various cell layers^46^. Therefore, we expect that enzymes of many other metabolic pathways will display similar divergence in their expression profiles when shifted from 2D monolayer to 3D microtumor growth environments, changes that potentially can be predicted by sophisticated metabolic models^46^.

A central aim of cancer metabolism research is to identify metabolic pathways selectively activated in tumor cells and thus, to uncover potential therapeutic targets. Hence, there are also potential practical consequences of our findings for the antimetabolic therapies of primary tumors and/or (micro)metastases. Previous experiments have shown that knockdown of PHGDH in breast cancer cells with amplified *PHGDH* copy numbers attenuates the proliferation of these cells without changes in intracellular serine levels and this effect cannot be rescued by exogenous serine^15^. Thus, PHGDH expression in the outer layer of growing microtumors may be required not for maintenance of serine levels but for maintaining this metabolic flux from a SGS pathway regulatory perspective that could affect the activity (and not the expression level) of other enzymes of the pathway. Indeed, PHGDH inhibitor compounds have been shown to reduce the incorporation of one-carbon units into nucleotides both from glucose and exogenous serine^47^ suggesting that glycolytic serine synthesis is required for coordinating the use of one carbon units from endogenous and exogenous serine in nucleotide synthesis. Given the highly restricted PHGDH expression in 3D microtumors, it may be that PHGDH inhibitors alone^47–49^ may have a more limited effect on delaying tumor growth in patients than in combination with other antimetabolic therapies.

Limitations of our study include the fact that we obtained only static enzyme expression data on mt150 and mt600 microtumors with only very limited information about the microenvironment that evolved within the growing microtumors. In other contexts, dynamics of protein expressions on a single-cell level have been examined, with or without concomitant tracing of transcriptome and secretory states^50–52^. Thus, in future studies, enzymes of the SGOC pathway and other metabolic pathways will need to be examined at high spatial and temporal resolution, in both mt150 and mt600 microtumors. Similarly, the regulatory mechanisms responsible for the observed enzyme expression patterns will need to be uncovered. Given that tumor metabolic activities are influenced by the tumors’ tissues of origin^38,39^, these studies will need to be performed in microtumors derived from various tumor types, both in the presence and absence of other tumor and non-tumor cell types. Similarly, the metabolic behavior of microtumors comprised of strictly epithelial tumor cells mixed together with those that are fully or partially mesenchymal (and thus, competent for metastasis initiation), and/or in the presence of cancer associated- or non-tumorigenic stromal fibroblast will be of major interest.

## Methods

### 2D monolayer cell cultures

Fourteen cancer cell lines from the NCI-60 panel^23^, including HCT-116, KM-12 (colon cancer), IGROV1, OVCAR3 (ovarian cancer), HS-578T, T47D (breast cancer), HOP-92, NCI-H322M (lung cancer), PC-3, DU-145 (prostate cancer), SK-MEL-5, MDA-MB-435 (melanoma), and SF-295, SF-539 (brain cancer), were cultured as 2D monolayers in RPMI 1640 medium (Life Technologies, Grand Island, NY), which contains both the amino acids serine and glycine, supplemented with 10% heat-inactivated fetal bovine serum (HI-FBS, Life Technologies) and 1% penicillin/streptomycin (Life Technologies) at 37 °C with 5% CO_2_.

### Western blotting

Western blot analysis was performed following our previously reported procedure^24^. The membranes were probed with monoclonal mouse antibodies, anti-PHGDH (1:50, Santa Cruz Biotechnology, Santa Cruz, CA) and anti-SHMT2 (1:500, Cell Signaling Technology, Beverly, MA) and monoclonal rabbit antibodies, anti-PSPH (1:100, Santa Cruz Biotechnology), anti-PSAT (1:250, Novus Biologicals, Littleton, CO), and anti-SHMT1 (1:250, Novus). As a loading control, a monoclonal mouse antibody to β-actin (1:500, Abcam Inc., Cambridge, MA) was used.

### Immunocytochemical analysis of 2D monolayers

For immunocytochemical analysis, cultured cells grown in a 12-well plate were fixed with 2% paraformaldehyde (Sigma-Aldrich, St. Louis, MO) for 30 min, washed in PBS and then treated with peroxidase block solution (DAKO, Carpinteria, CA) to inactivate endogenous peroxidase activity. Following a PBS wash, cells were permeabilized with 0.1% Triton-X-100 (Fisher Scientific, Pittsburgh, PA) and non-specific proteins were blocked in 2% BSA for 15 min at room temperature (RT). This step was followed by incubation in a humidified atmosphere at 37 °C for 1 hour with primary monoclonal mouse antibody to PHGDH (Santa Cruz Biotechnology) diluted 1:15 in PBS. After washing the cells with PBS, the immunocytochemical analysis for PHGDH was conducted for 30 min at RT using DAKO EnVision + System-HRP. After washing the cells with PBS again, the immunoreactions were visualized with 3,3′-diaminobenzidine (DAB) solution (DAKO). Images were captured with the 40X objective lens on the TS100 microscope (Nikon, Tokyo, Japan). Negative controls, in which the primary antibodies were replaced with PBS and non-immune sera, did not show nonspecific staining.

### Immunofluorescence staining and microscopy of 2D monolayers

Immunofluorescence staining was performed according to our previous protocol^24^. Briefly, the cells were incubated with primary antibodies: monoclonal mouse antibody to PHGDH (1:50, Santa Cruz Biotechnology) and SHMT2 (1:80, Abcam), and monoclonal rabbit antibodies to PSPH (1:50, Santa Cruz Biotechnology), PSAT (1:100, Novus), SHMT1 (1:80, Novus), TOM20 (1:100, Abcam), and Ki-67 (1:400, Abcam) in a humidified atmosphere for 1 hour at 37 °C. As secondary antibodies, Alexa Fluor 488 goat anti-mouse IgG and Alexa Fluor 555 goat anti-rabbit IgG were used (both; 1:200, Abcam). Nuclei were stained with Hoechst (50 μg/ml). Images were captured with the 40X oil objective lens on the Olympus Provis fluorescence microscope (Olympus Optical, Tokyo, Japan).

### 3D culture and microtumor fabrication

Microtumors of DU-145 cells were fabricated using non-adhesive hydrogel arrays with 150 and 600 µm size microwells^18,20^. Briefly, hydrogel microwell arrays were prepared *in-house* using polyethylene glycol dimethacrylate (PEGDMA, 1000 Da, Sigma-Aldrich) and polydimethyl siloxane (PDMS, Dow) molds. Microwells of sizes 150 and 600 µm patterned on silicon masters were used to generate defined size PDMS posts. PDMS molds with posts were first fabricated, as described earlier^18,20^, and thereafter, were used for making hydrogel microwell devices. PDMS posts were then placed on PEGDMA solution mixed with photo-initiator (Irgacure-1959, 1% w/w, Ciba AG, Basel, Switzerland) and photo-crosslinked using the OmniCure S2000 curing station (EXFO, Mississauga, Canada). PDMS stamp was then peeled from the substrate to obtain hydrogel microwell devices.

The metastatic prostate cancer-derived DU-145 cell line was used to generate mt150 and mt600 microtumors. The DU-145 cell line was maintained in RPMI containing 10% FBS and 0.1% penicillin/streptomycin. Cells were grown to attain confluency of 40‒60% and used for 3D microtumor fabrication. Fifty microliter of cell suspension (1 × 10^6^ cells/device) was seeded on the 1 cm^2^ hydrogel microwell device. Cells were allowed to settle in the microwells by gravity. Undocked cells were washed gently with PBS 2‒3 times and cultured in a humidified 5% CO_2_ incubator at 37 °C. Uniform size microtumors were cultured in their respective media and harvested on day 4 and processed further for immunostaining.

### Immunostaining and confocal microscopy of 3D microtumors

Microtumors were fixed with 4% paraformaldehyde (Sigma-Aldrich) for 30 min at RT. Subsequently, they were washed with phosphate buffered saline with Tween 20 (PBST) to remove residual PFA and again fixed with 95% methanol on ice for 15 min at −20 °C. Permeabilization was done with 0.1% Triton X-100 for 90 min followed by further blocking with 3% BSA to avoid non-specific binding. Microtumors were incubated with primary antibodies for SHMT1 (1:100), SHMT2 (1:100), PHGDH (1:100), PSAT (1:100), PSPH (1:100), TOM-20 (1:100) and Ki-67 (1:100) diluted in blocking buffer overnight at 4 °C in a humidified chamber along with a negative control (without primary antibody). After washing thrice with PBST, microtumors were incubated with fluorescently labeled secondary antibodies overnight at 4 °C. Nuclei were stained by incubating with Hoechst (1:500) at 4 °C overnight. Stained microtumors were kept in glycerol at 4 °C until confocal imaging. Images were acquired on a confocal microscope Olympus Fluoview (Olympus Optical) as z-stack containing a series of 5 µm slices using 20X or 40X objectives. The images are presented as 2D projection of maximum intensity.

### Compartmental model and simulations

Using the BioNetGen language^53^ we developed a mathematical model that describes protein expression, nutrient abundance, and transport of molecules between compartments of a 3D microtumor. The simulated microtumor consists of four compartments: a core with 300 µ radius, an inner layer shell between 300–450 µ, an outer layer shell of 450–600 µ radius, in addition to an extracellular compartment (Fig. 6A). The model was simulated deterministically, using ordinary differential equations derived from the rules of the model described below. Simulations were run for 600,000 time units, which was sufficient for the system to reach steady-state values for molecule numbers in each compartment. The input file for the model is provided in the supplementary data file (MicrotumorModel.BNGL).

The complete set of molecular interactions and other processes in the model were represented through 26 reaction rules and 31 reaction rate constants (summarized in table S1). The reaction network consists of four molecule types that include nutrients (NU), a class of genes associated with proliferation (‘Ki67′), the rate-limiting enzyme in serine biosynthesis (PHGDH), and a hypothetical inhibitor (Inh) that behaves as the aggregate of stress-inducible factors that limit the expression of PHGDH. The model assumes that growth medium in the extracellular compartment provides a constant source of NU, which can diffuse into the outer layer and throughout the microtumor (Fig. 6B). Rate constants for flux of NU diffusion between the core and inner layer compartments were adjusted to account for differences in their respective surface areas. Nutrients are consumed in each compartment by the production of ‘Ki67′, Inh, in addition to PHGDH. In each compartment, NU is also produced through the action of PHGDH to model *de novo* biosynthesis. ‘Ki67′ and PHGDH are confined to the compartment in which they are expressed, but Inh could transport between microtumor compartments. Expression of Inh is omitted in model M1, and in model M2 Inh was produced at a constant rate in the core (see table S1 for details). Seed concentrations within the three microtumor compartments were set to 0 for all molecule types.

### Data availability

All data generated or analysed during this study are included in this published article (and its Supplementary Information files)

## Electronic supplementary material

Supplementary Information

MicrotumorModel
