# Supplementary material for: Shift from stochastic to spatially-ordered expression of serine-glycine synthesis enzymes in 3D microtumors
Source: Sci Rep. 2018 Jun 20;8:9388. doi: 10.1038/s41598-018-27266-8 (PMC6010463; doi:10.1038/s41598-018-27266-8)
Supplement: Supplementary file 1 — Supplementary Information [file 41598_2018_27266_MOESM1_ESM.pdf]

<u>**SUPPLEMENTARY INFORMATION**</u>

Shift from stochastic to spatially-ordered expression of serine-glycine synthesis enzymes in 3D microtumors

Manjulata Singh[a,¶], Tomoko Warita[b,¶], Katsuhiko Warita[b], James R. Faeder[c], Robin E.C. Lee[c,1], Shilpa Sant[a,d,1] and Zoltán N. Oltvai[b,c,1]

[a] Department of Pharmaceutical Sciences, School of Pharmacy, University of Pittsburgh, Pittsburgh, PA 15261, USA

[b] Departments of Pathology and [c] Computational & Systems Biology, School of Medicine, University of Pittsburgh, Pittsburgh, PA 15213, USA

[d] Department of Bioengineering, Swanson School of Engineering, McGowan Institute for Regenerative Medicine, and UPMC-Hillman Cancer Center, University of Pittsburgh, Pittsburgh, PA 15261, USA

Supplementary Table 1 p.2

Supplementary Figures p.3-13

Supplementary Results p.14-17

Table S1 Reaction rules included in the model with diffusion-normalized rate constants. For reversible rules the forward rate constant is followed by the reverse rate. Subscripts denote extracellular (e), outer layer (o), inner layer (i), and core (c) compartments.

Reaction rule	Rate	Description
0→NUo	0.00001*NUe	import of nutrients from extracellular compartment (produced in proportion with NUe)
NUo→ 0	0.0001	export of nutrients from the outer layer to the extracellular compartment (modeled as degradation)
NUo→PHGDHo	0.001/(1+5*INHIBo)	PHGDH production in the outer layer (nutrients are consumed
NUi→PHGDHi	0.001/(1+5*INHIBi)	PHGDH production in the inner layer (nutrients are consumed
NUc→PHGDHc	0.001/(1+5*INHIBc)	PHGDH production in the core layer (nutrients are consumed
NUo→Ki67o	0.1	'Ki67' production in the outer layer (nutrients are consumed
NUi→Ki67i	0.1	'Ki67' production in the inner layer (nutrients are consumed
NUc→Ki67c	0.1	'Ki67' production in the core layer (nutrients are consumed
NUc→INHIBc	M1: 0<br>M2: 0.05	inhibitory molecule production in the core (nutrients are consumed)
INHIBc↔INHIBi	0.001,0.001	inhibitory molecule transport between the core and the inner layer
INHIBi↔INHIBo	0.001,0.001	inhibitory molecule transport between the inner layer and outer layer
INHIBo↔INHIBe	0.001,0.001	inhibitory molecule transport between the outer layer and extracellular compartment
NUo↔NUi	1,1	Nutrient diffusion between outer and inner layers
NUi↔NUc	1/2.25; 1/2.25	Nutrient diffusion from the inner layer to core; diffusion rate accounts for surface area of core
PHGDHo→NUo+PHGDHo	1	*de novo* biosynthesis of nutrients in the outer layer
PHGDHi→NUi+PHGDHi	1	*de novo* biosynthesis of nutrients in the inner layer
PHGDHc→NUc+PHGDHc	1	*de novo* biosynthesis of nutrients in the core
Ki67o,i,c→0	0.01	Degradation of Ki67 (same in each compartment)
PHGDHo,i,c→0	0.05	Degradation of PHGDH (same in each compartment)
INHIBo,i,c→0	0.005	Degradation of inhibitory molecule (same in each compartment)

Supplementary Figures

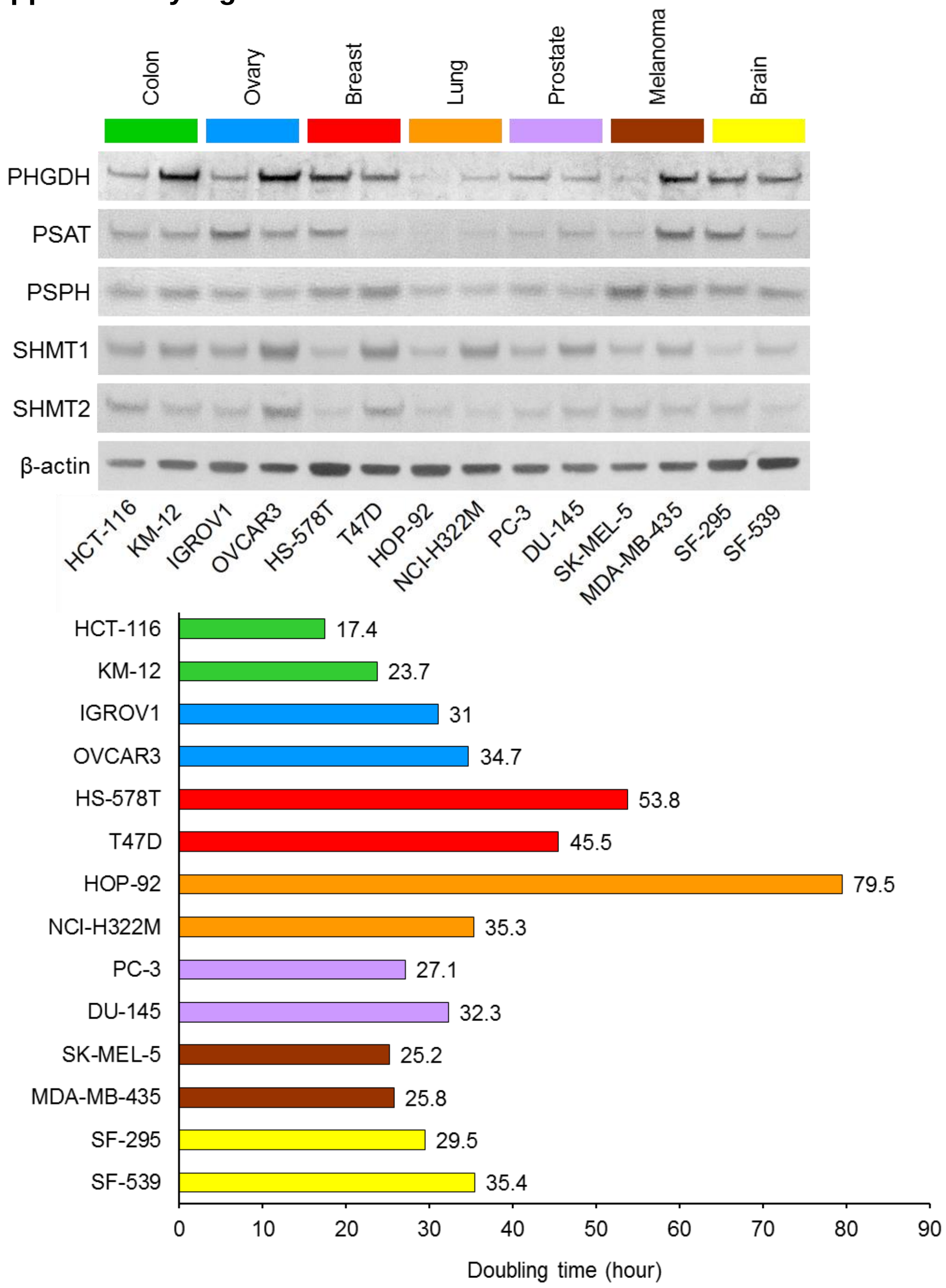

Figure S1: Average SGS enzyme expression level vs. cell line doubling time

Average serine-glycine synthesis (SGS) enzyme expression vs. doubling time of cell lines. Data for the latter were from https://dtp.cancer.gov/discovery_development/nci-60/ cell_list.htm. While rapidly proliferating cell lines (short doubling time) tend to have PHGDH expression higher than in slowly proliferating ones, there is no direct correlation between the two values.

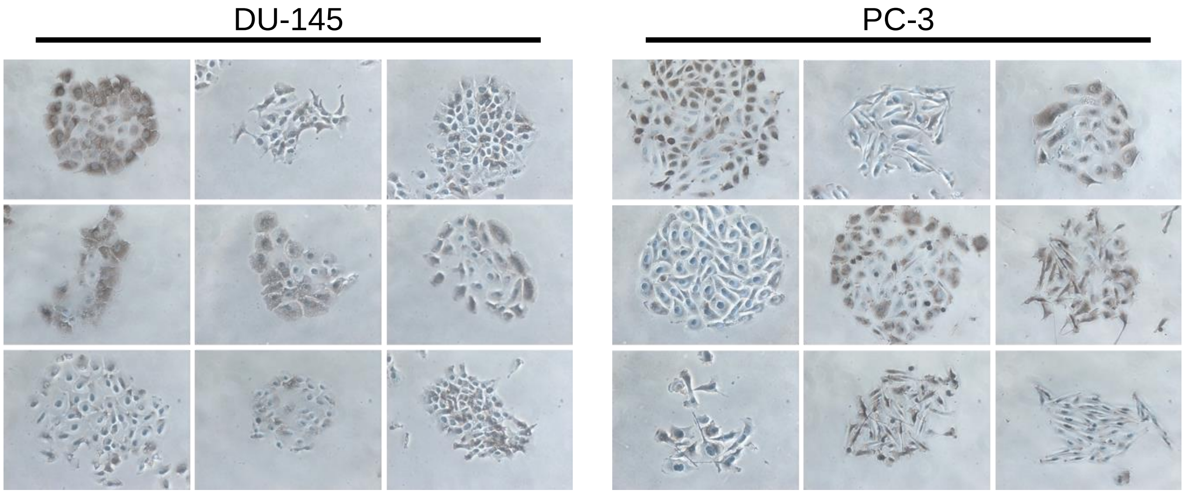

Figure S2: PHGDH expression in single cell-derived clones of cell lines

PHGDH immunoreactivity of single cell-derived clones of DU-145 (left panels), and PC-3 cell lines (right panels). Each image represents different colonies derived from individual clones of the same cell line. We used 3,3'-diaminobenzidine (DAB) for visualization.

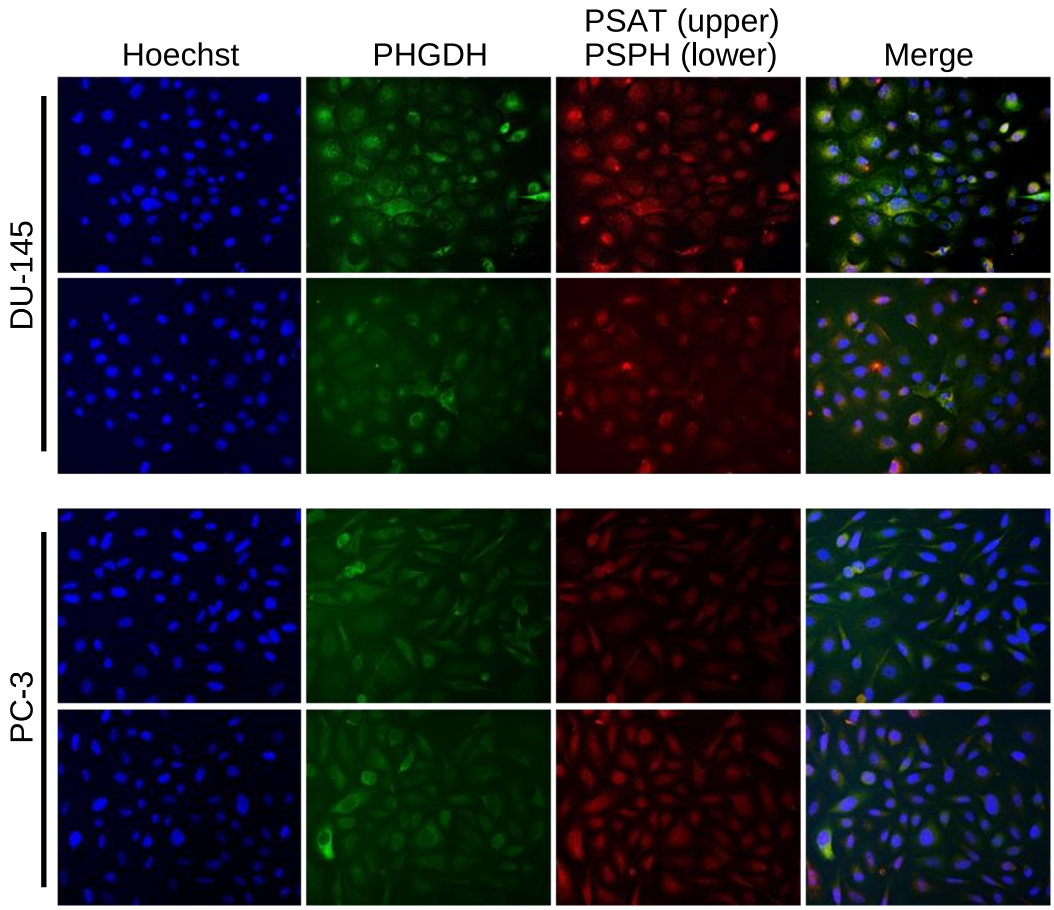

Figure S3: Expression of serine synthesis enzymes in DU-145 and PC-3 cell lines

Phosphoglycerate dehydrogenase (PHGDH) (green channels), and phosphoserine amino-transferase (PSAT) (red channel, top panel) or phosphoserine phosphatase (PSPH) (red channels, bottom panel) immunoreactivity in DU-145 and PC-3 prostate cancer cell lines are shown. Hoechst counterstain (blue color) indicates cell nuclei.

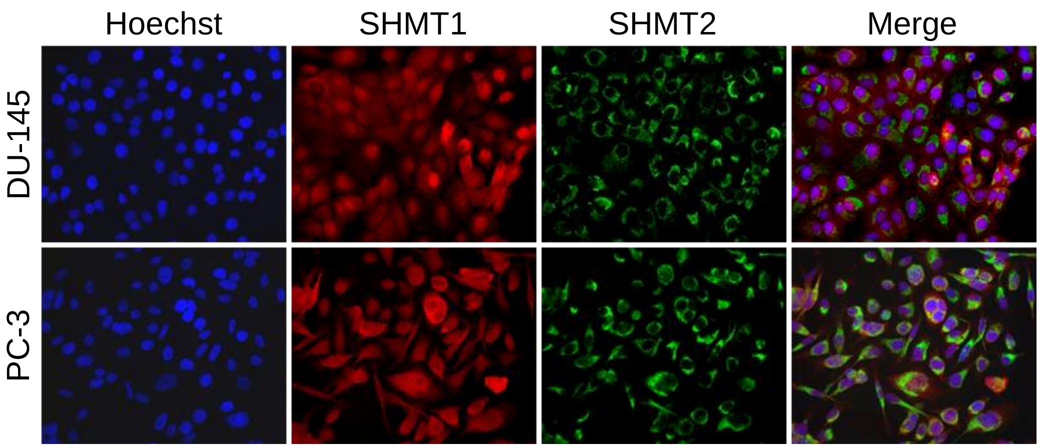

Figure S4 Expression of glycine synthesis enzymes in DU-145 and PC-3 cell lines

Cytoplasmic serine hydroxymethyltransferase 1 (SHMT1) (red channel), and mitochondrial SHMT2 (green channel) immunoreactivity are shown in DU-145 and PC-3 prostate cancer cell lines. Hoechst counterstain (blue color) indicates cell nuclei.

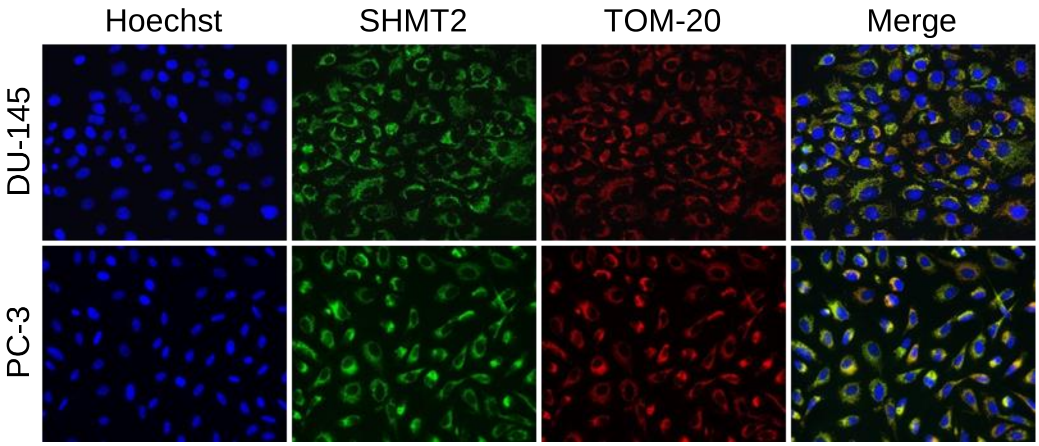

Figure S5 Expression of mitochondrial SHMT2 and TOM-20 in DU-145 and PC-3 cell lines

Mitochondrial serine hydroxymethyltransferase 2 (SHMT2) (green channel), and mitochondrial marker protein, TOM-20 (red channel) immunoreactivity are shown in DU-145 and PC-3 prostate cancer cell lines, demonstrating co-localization. Hoechst counterstain (blue color) indicates cell nuclei.

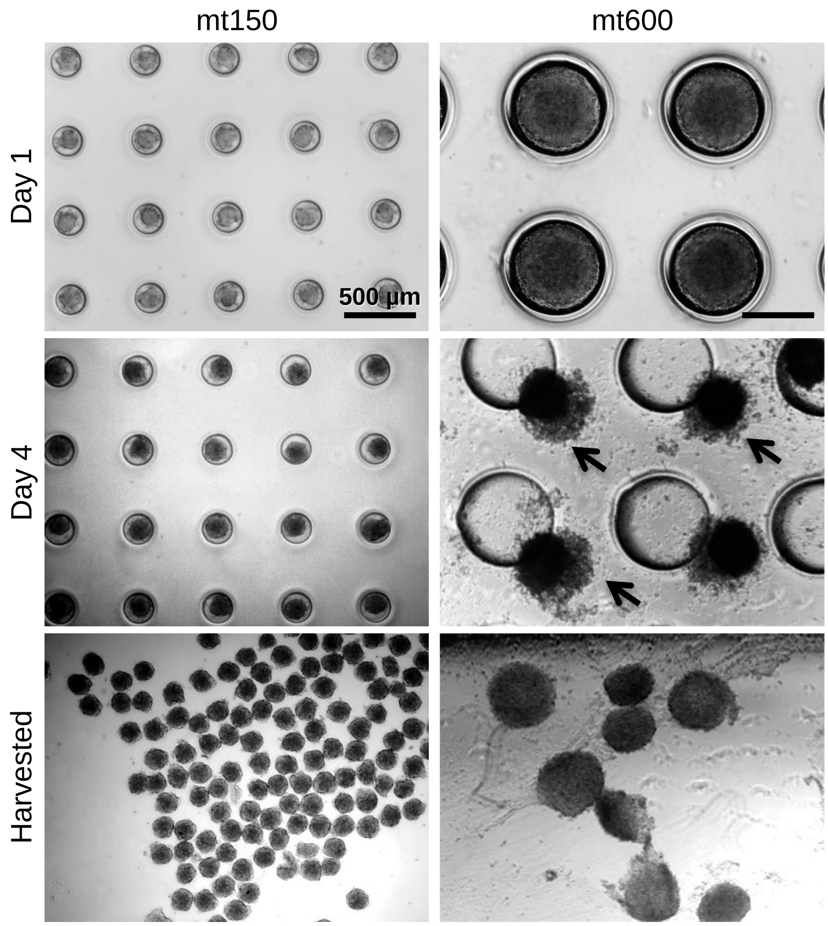

Figure S6: Size-controlled 3D microtumors of DU-145 tumor cells.

Photomicrographs showing microtumors (mt150 and mt600) on day 1 (top panel) and day 4 (middle panel) on hydrogel microwell devices. Interestingly, mt600 showed collective migration of the cells out of the wells on day 4 (black arrows). Microtumors were harvested by gentle flushing of the hydrogel device on day 4. Harvested microtumors (bottom panel) demonstrated uniform size microtumors.

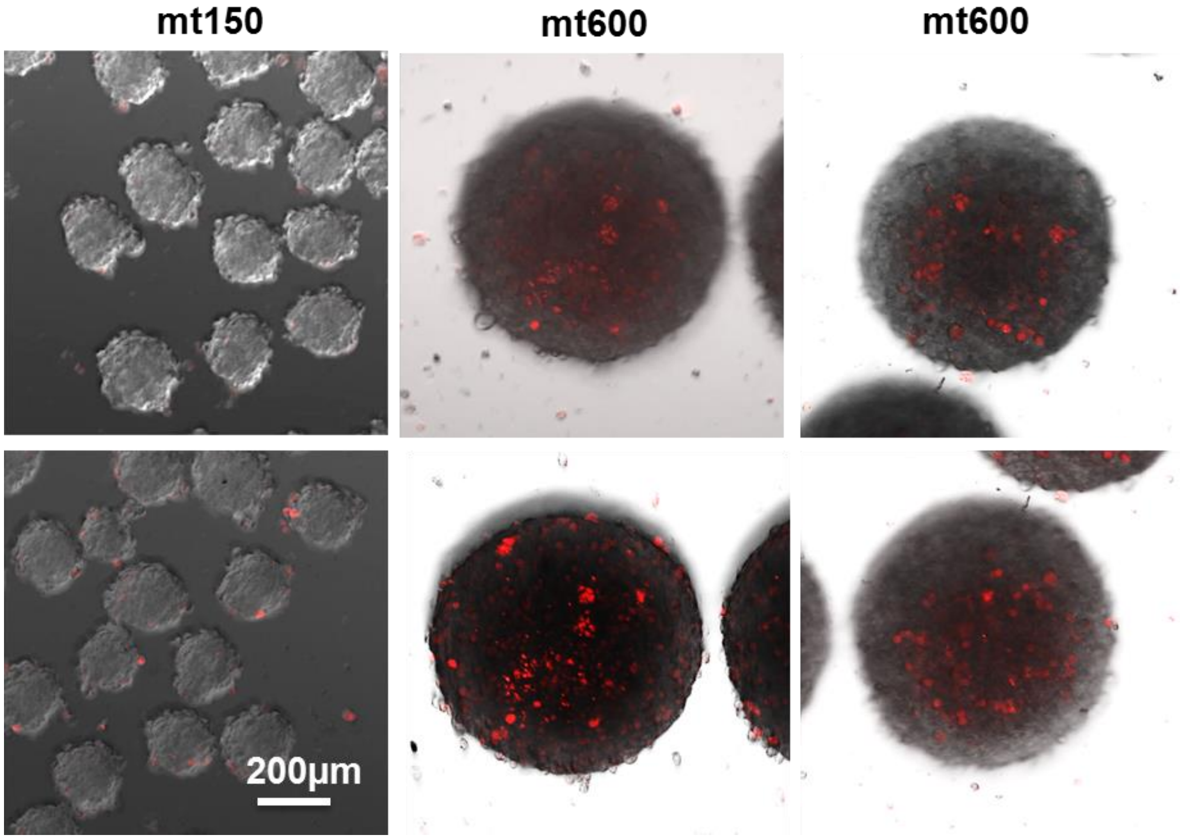

Figure S7 Oxygenation levels in 3D microtumors

Oxygenation levels in DU-145-derived microtumors as determined by the hypoxia sensitive dye, Ru-dpp (red). Hypoxic cells (red) in the interior of mt600 but not mt150 microtumors are evident. Multiple microtumors of both mt600 but not mt150 are shown.

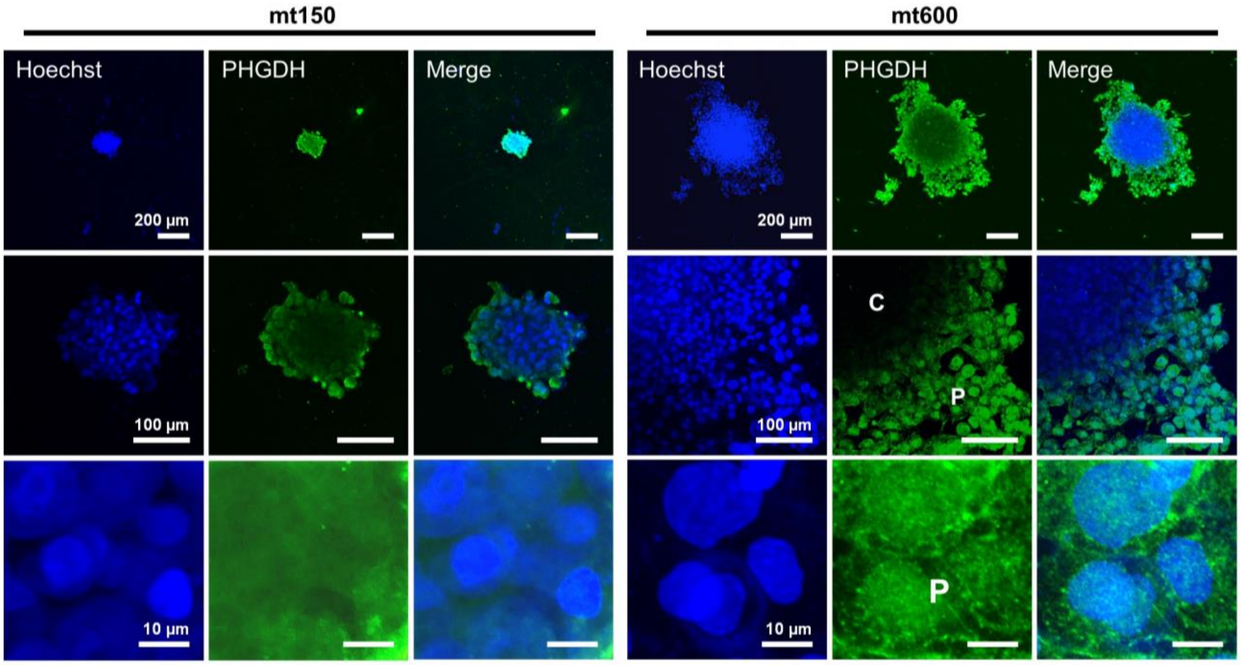

Figure S8: Expression of PHGDH in 3D microtumors

Cellular localization of PHGDH (green) in DU-145 derived mt150 and mt600 microtumors at low- (top panels), intermediate- (middle panels) and high magnification (bottom panels). Central (C) and peripheral (P) areas of the microtumors are denoted. In the overlay panels, Hoechts counterstain (blue color) indicates cell nuclei. PHGDH expression in both mt150 and mt600 is localized selectively to the most peripheral cell layers of the microtumors.

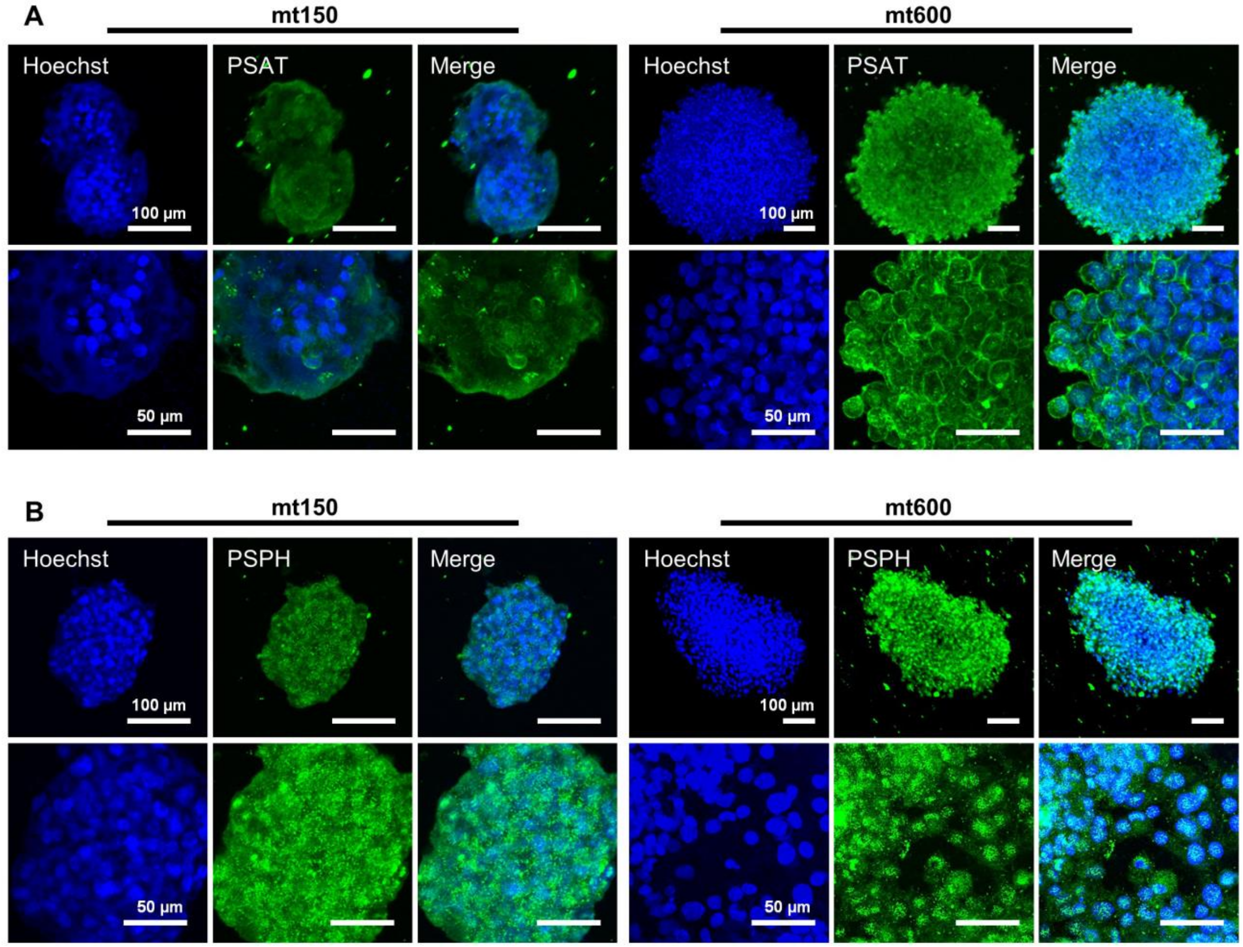

Figure S9: Expression of PSAT and PSPH in 3D microtumors

Subcellular localization of (A) PSAT and (B) PSPH (green) in mt150 and mt600 DU-145-derived microtumors at low- (top panels) and high magnification (bottom panels). PSPH displays uniform expression in both mt150 and mt600, while PSAT expression distribution is intermediate between PHGDH-like or PSPH-like staining patterns.

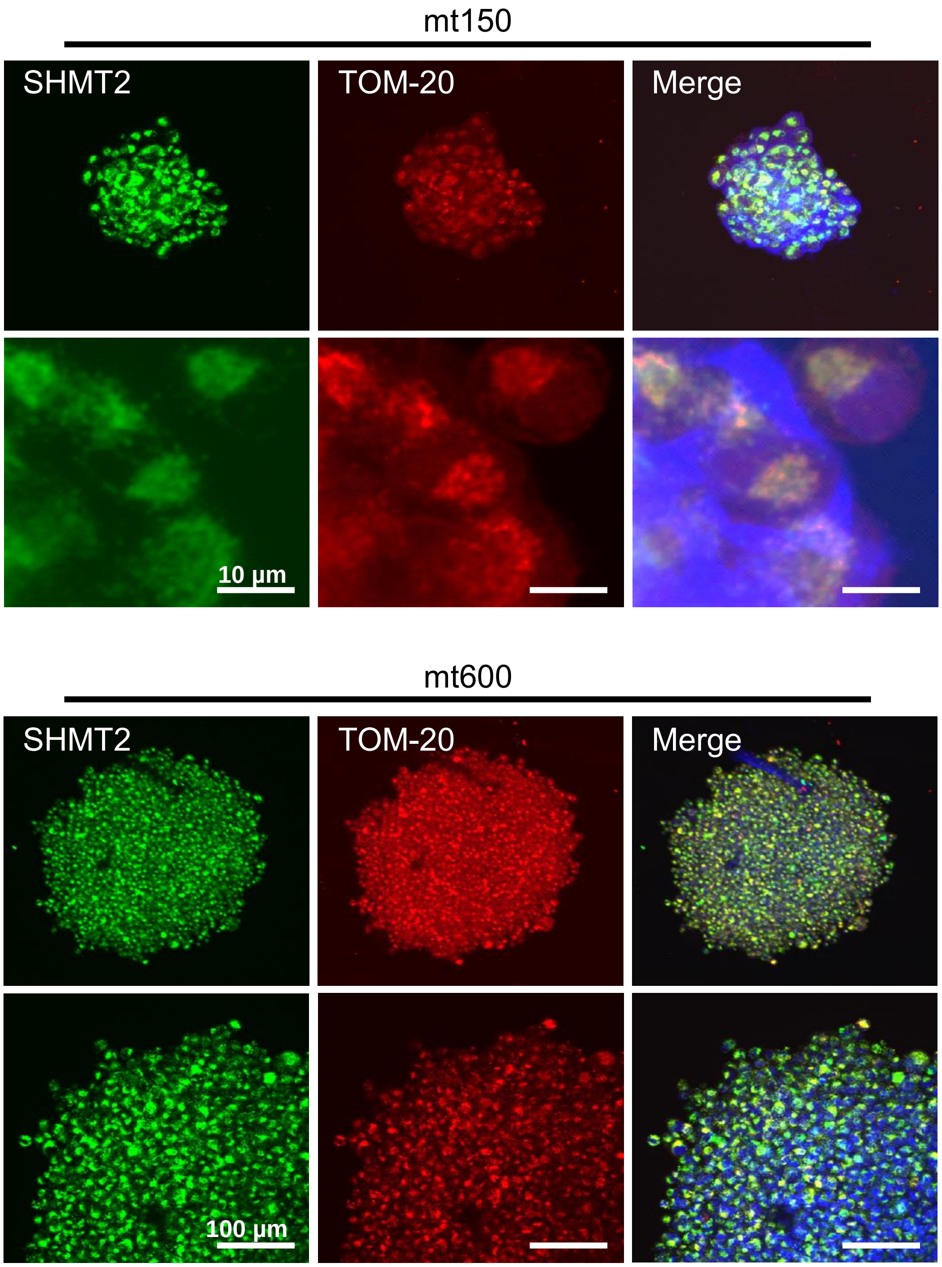

Figure S10 Mitochondrial expression of SHMT2 in 3D microtumors

Spatial localization of SHMT2 (green) and TOM-20 (red) proteins in DU-145 derived mt150 and mt600 at low- (top panel) and high magnification (bottom panels). In the overlay panels, Hoechst counterstain (blue color) indicates cell nuclei. SHMT2 expression co-localizes with that of the mitochondrial marker protein, TOM-20, confirming its mitochondrial localization.

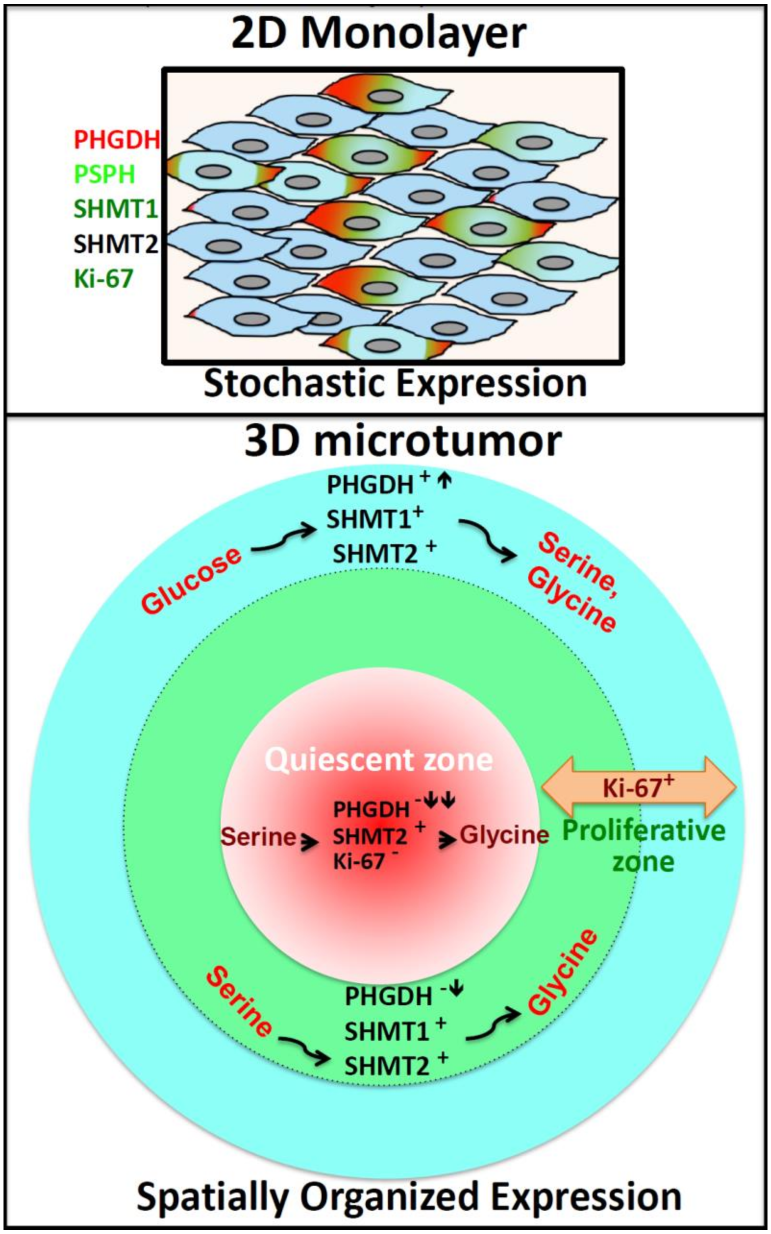

Figure S11 Model of environment-induced metabolic switch

In 2D-cell monolayers (top panel) where all cells encounter an essentially identical extracellular environment there is a stochastic expression of several metabolic enzymes, such as PHGDH or PSPH. However, in 3D microtumors cells are surrounded by various microenvironments to which they probably respond directly and through cell-cell signaling. In our case, the spatial localization of SG pathway enzyme expression within mt150 and mt600 3D microtumors identifies a $PHGDH^{+}$, serine synthesizing outer proliferative ($Ki\text{-}67^{+}$) layer, followed by a proliferative layer with SHMT1 and SHMT2-driven serine-glycine conversion and a deeper, non-proliferative layer with seemingly dominant, mitochondrial SHMT-2 driven serine-glycine conversion. As there is no evidence for hypoxia within the smaller, mt150 microtumors, glucose-derived serine synthesis in the outermost rapidly proliferating cell layers is likely required for coordinating the use of one-carbon units from endogenous and exogenous serine in nucleotide synthesis.

Supplementary Results

Effect of permeabilization protocols on immunofluoresecence staining

To select the optimal cell permeabilization protocol to be used throughout the study, in preliminary experiments we compared immunofluorescence results following cell permeabilization with 0.1% Tween 20 or Triton X-100. Cultured cells grown on coverslips in a 24-well plate were fixed with 2% paraformaldehyde (Sigma-Aldrich, St. Louis, MO) for 30 min, washed in PBS, and then permeabilized with 0.1% Tween-20 or 0.1% Triton-X-100 (Fisher Scientific, Pittsburgh, PA) made in PBS for 15 min. Following a PBS wash, non-specific proteins were blocked in 2% BSA for 15 min at RT. The cells were incubated with primary antibodies: monoclonal mouse antibody to PHGDH (1:50, Santa Cruz Biotechnology, Santa Cruz, CA) and SHMT2 (1:80, Abcam Inc., Cambridge, MA), and monoclonal rabbit antibodies to PSPH (1:50, Santa Cruz Biotechnology), PSAT (1:100, Novus Biologicals, Littleton, CO), SHMT1 (1:80, Novus), TOM-20 (1:100, Abcam), and Ki-67 (1:400, Abcam) in a humidified atmosphere for 1 hour at 37°C. Coverslips were then probed with Alexa Fluor 488 goat anti-mouse IgG and Alexa Fluor 555 goat anti-rabbit IgG (both; 1:200, Abcam) in the dark for 15 min at RT. Following a PBS wash, nuclei were stained with Hoechst 33342 (50 μg/ml) for 5 min at RT, washed and mounted in an aqueous-based mounting medium Clearmount™ (Invitrogen, Carlsbad, CA). Images were captured with the 40X oil objective lens on the Olympus Provis fluorescence microscope (Olympus Optical, Tokyo, Japan).

Figure SR1: Comparison of serine synthesis enzyme stainings (Tween 20 vs. Triton X-100)

Figure SR2: Comparison of SHMT2 and TOM20 enzyme stainings (Tween 20 vs. Triton X-100)

Figure SR3: Comparison of nuclear Ki-67 staining (Tween 20 vs. Triton X-100)

In all pilot experiments permeabilization of the cell mitchondria and nuclei with Triton X-100 proved much better than using Tween20. Based on these results we elected to use 0.1% Triton X-100 permeabilization in all experiments reported in the main text.

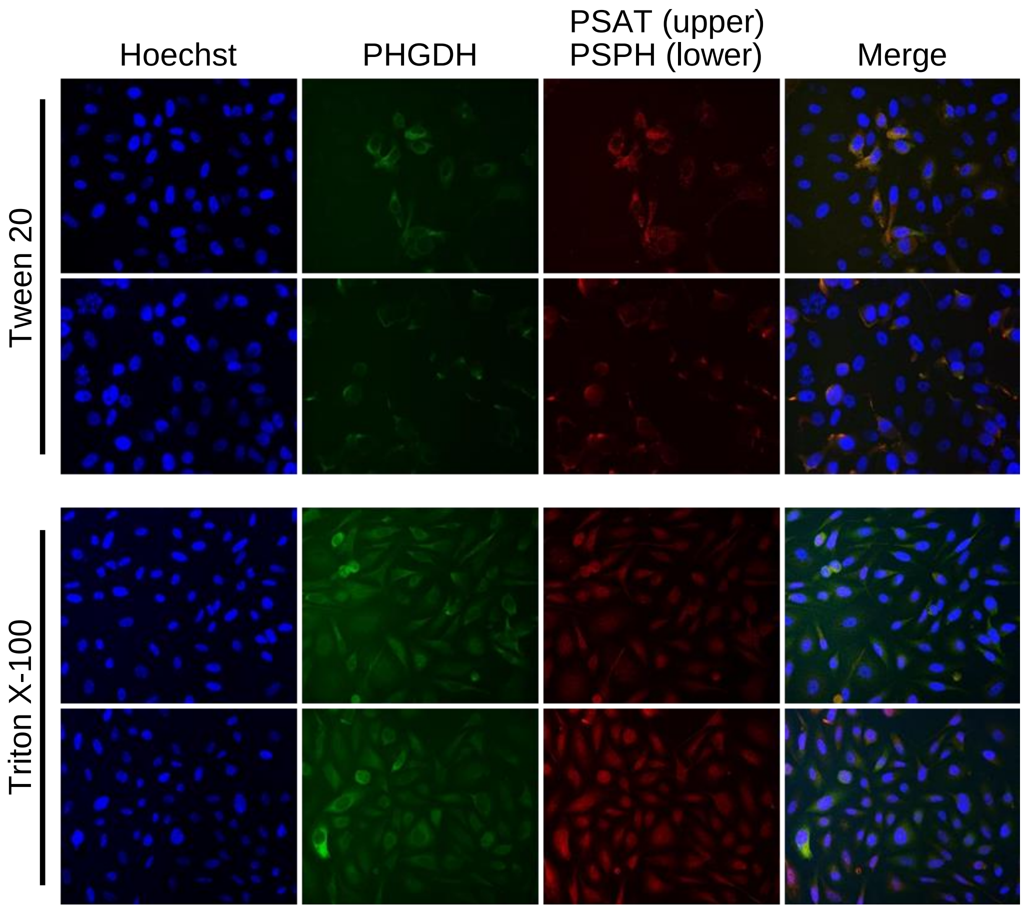

Figure SR1 Co-expression of serine synthesis enzymes in PC-3 cells with two different cell permeabilization protocols.

Phosphoglycerate dehydrogenase (PHGDH) (green channels), and phosphoserine amino-transferase (PSAT) (red channel, top panel) or phosphoserine phosphatase (PSPH) (red channels, bottom panel) immunoreactivity in the PC-3 prostate cancer cell line are shown. Hoechst counterstain (blue color) indicates cell nuclei. It is evident that the gentler permeabiliation protocol (Tween 20) results in fewer cells being permeabilized but the staining pattern remains exclusively cytoplasmic. When using the stronger detergent (Triton X-100) some non-cytoplasmic staining pattern has also been seen that may be due to detergent-induced neoepitopes or detergent-induced cell death/ apoptosis.

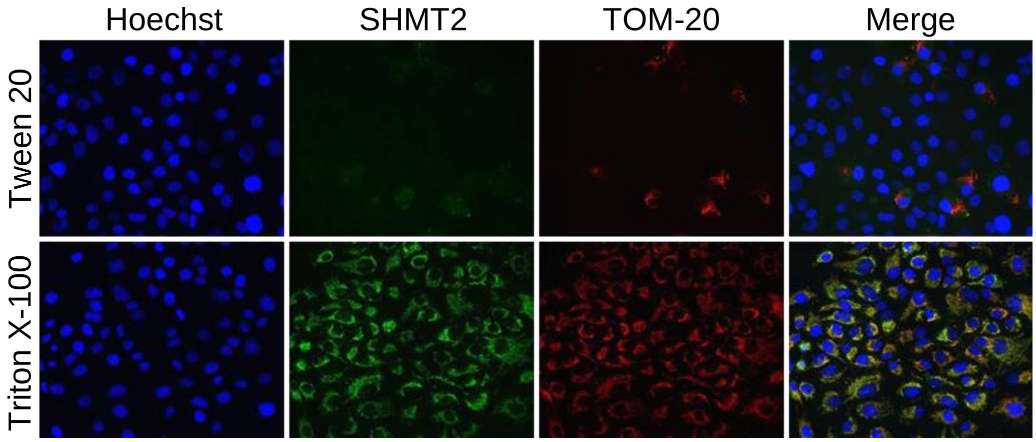

Figure SR2 Co-expression of glycine synthesis enzyme SHMT2 and mitochondrial marker protein TOM-20 in DU-145 cells with two different permeabilization protocols.

Mitochondrial serine hydroxymethyltransferase 2 (SHMT2) (green channel), and mitochondrial marker protein, TOM-20 (red channel) immunoreactivity are shown in the DU-145 prostate cancer cell lines, demonstrating co-localization. Hoechst counterstain (blue color) indicates cell nuclei. It is evident that Triton X-100 allows the antibody to access to mitochondrial proteins more effectively than Tween 20.

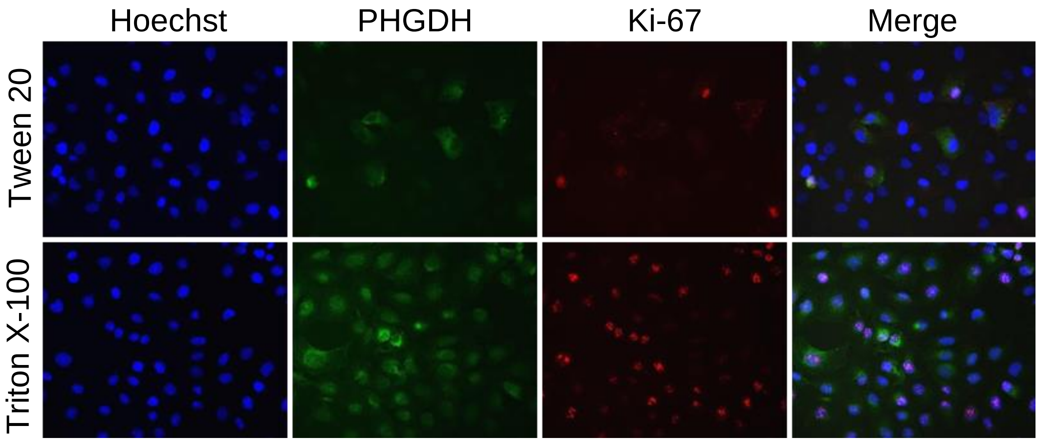

Figure SR3 Co-expression of PHGDH and nuclear Ki-67 in DU-145 cells with two different permeabilization protocols.

Phosphoglycerate dehydrogenase (PHGDH) (green channels), and nuclear Ki-67 (red channel) immunoreactivity in the DU-145 prostate cancer cell line are shown. Hoechst counterstain (blue color) indicates cell nuclei. It is evident that Triton X-100 provides more effective access of the antibodies to cytoplasmic antigen PHGDH and to intranuclear antigen Ki-67 than Tween 20.
